# The association of arterial partial oxygen pressure with mortality in patients with severe acute pancreatitis: a retrospective cohort study

**DOI:** 10.1186/s40635-025-00843-8

**Published:** 2025-12-18

**Authors:** Yiji Chen, Jianhua Wan, Wenqing Shu, Xiaoyu Yang, Huajing Ke, Wenhua He, Yin Zhu, Nonghua Lu, Liang Xia

**Affiliations:** Department of Gastroenterology, Jiangxi Provincial Key Laboratory of Digestive Diseases, Jiangxi Clinical Research Center for Gastroenterology, Digestive Disease Hospital, The First Affiliated Hospital, Jiangxi Medical College, Nanchang University, 17 Yongwaizheng Street, Nanchang, 330006 Jiangxi PR China

**Keywords:** Severe acute pancreatitis, Oxygen, Intensive care unit, Acute hypoxemic respiratory failure

## Abstract

**Background:**

Patients with severe acute pancreatitis (SAP) frequently develop hypoxic acute respiratory failure (AHRF), with a mortality rate as high as 37%. However, the optimal partial pressure of oxygen (PaO_2_) for SAP patients remains unclear to date. This study aims to investigate whether partial pressure of oxygen is associated with mortality in SAP patients.

**Methods:**

A retrospective cohort study was conducted on patients with severe acute pancreatitis (SAP) admitted to the First Affiliated Hospital of Nanchang University from 2015 to 2024. Propensity score matching (based on whether arterial oxygen partial pressure PaO_2_ ≥ 80 mmHg during the first 3 days after ICU admission, assigning patients to the liberal PaO_2_ group or conservative PaO_2_ group), univariate logistic regression analysis, Cox regression analysis, subgroup analysis, Kaplan–Meier (K–M curve) survival analysis, and sensitivity analysis were employed to thoroughly evaluate the association between PaO_2_ and mortality in SAP patients. The primary outcome was 28-day mortality.

**Results:**

The study included 1585 patients. We found that higher PaO_2_ was associated with lower 28-day mortality rates. In logistic regression analysis after propensity score matching, the incidence rates of adverse outcomes such as persistent circulatory failure (OR 0.50; 95% CI 0.35–0.69; *P* < 0.001) and persistent multiple organ failure (OR 0.60; 95% CI 0.47–0.78; *P* < 0.001) significantly decreased. The K–M curve demonstrated significant reductions in 28-day mortality (*P* = 0.02), 90-day mortality (*P* = 0.0079), and overall mortality (*P* = 0.008) in the liberal PaO_2_ group, with all *P* values showing statistical significance. Subgroup analysis revealed that the association between higher PaO_2_ and mortality in SAP patients varied across different age groups, BMI values, SIRS and APACHE II scores, and smoking status. Sensitivity analysis demonstrated stable results after excluding specific populations. On the third day of ICU admission (*P* = 0.016), higher PaO_2_ correlated with improved outcomes compared to the conservative group, particularly when PaO_2_ stabilized around 100 mmHg.

**Conclusions:**

Early maintenance of higher PaO_2_ (≥80 mmHg) during the initial ICU period was associated with lower mortality.

**Supplementary Information:**

The online version contains supplementary material available at 10.1186/s40635-025-00843-8.

## Introduction

Approximately 20–30% of patients with acute pancreatitis (AP) progress to severe acute pancreatitis (SAP). We typically confirm diagnoses based on the 2012 revised International Atlanta classification Criteria. The prognosis of SAP depends not only on pancreatic injury itself but is also closely associated with persistent organ failure, among which acute hypoxemic respiratory failure (AHRF) represents a key factor contributing to early mortality [1]. The pathological mechanisms primarily involve microcirculatory dysfunction, impaired mitochondrial oxygen utilization, and damage to the alveolar–capillary barrier due to an excessive inflammatory response. Current guidelines recommend that during respiratory support in the intensive care unit (ICU), a partial pressure of PaO_2_ around 60 mmHg—corresponding to an oxygen saturation above 90%—should be maintained, which appears clinically reasonable [2, 3]. However, in SAP patients complicated by AHRF, this target may be insufficient to meet their high metabolic oxygen demand, potentially leading to serious complications and poor clinical outcomes [4, 5].

Previous studies have shown heterogeneous oxygenation targets across different diseases. For instance, de Jonge et al. reported a linear relationship between FiO_2_ and ICU mortality, along with a U-shaped association for PaO_2_ (i.e., both low and high PaO_2_ values were associated with increased mortality) [6]. Subsequent studies have further explored and refined these targets. Girardis et al. demonstrated that a conservative oxygenation strategy (maintaining PaO_2_ between 70 and 100 mmHg) was associated with reduced ICU mortality compared with conventional therapy; in contrast, Barrot et al. found that among patients with acute respiratory distress syndrome (ARDS), a conservative oxygenation strategy targeting PaO_2_ between 55 and 70 mmHg did not improve 28-day survival [7–9]. Although these studies provide a theoretical basis for oxygen management, the optimal PaO_2_ target specifically for patients with severe acute pancreatitis remains undetermined. Therefore, this study aims to investigate whether higher PaO_2_ (≥80 mmHg) is associated with lower mortality in patients with SAP.

## Methods

### Study design and participants

The study was approved by the ethics committee of The First Affiliated Hospital of Nanchang University (No. 2011001). Informed consent by verbal was obtained from all participants approved by the ethics committee. This study adopted a retrospective cohort study design. The research data were derived from patients with severe acute pancreatitis (*n* = 2625) admitted to the Pancreatic ICU Ward of the Department of Gastroenterology, the First Affiliated Hospital of Nanchang University, from 2015 to 2024. The exclusion criteria were as follows: (1) excluding patients under 18 years (*n* = 33); (2) excluding pregnant patients (*n* = 69); (3) excluding patients with end-stage liver and kidney diseases (*n* = 11); (4) excluding patients admitted to the ICU for less than 3 days (*n* = 112); and (5) excluding patients without PaO_2_ data for 3 consecutive days (*n* = 815). Finally, 1585 SAP patients were included. This manuscript was prepared according to the STROBE guidelines [10].

### Data collection and oxygen partial pressure grouping

Data were extracted from the electronic medical record system. Demographic variables included age, sex, and body mass index (BMI). Disease-related information encompassed smoking status, alcohol use, and medical history, including hypertension, diabetes, and chronic obstructive pulmonary disease (COPD). Clinical signs and symptoms included body temperature, heart rate, respiratory rate, and mean arterial pressure (MAP). Disease severity was assessed using the Systemic Inflammatory Response Syndrome (SIRS) criteria and Acute Physiology and Chronic Health Evaluation II (APACHE II) score. Laboratory parameters consisted of white blood cell count (WBC), hematocrit (HCT), albumin (ALB), triglycerides (TG), blood urea nitrogen (BUN), creatinine (Cr), serum calcium (Ca), pH, Inspired Oxygen concentration (FiO_2_). Complications recorded included acute peripancreatic fluid collection (APFC), pancreatic pseudocyst, walled-off necrosis (WON), acute necrotic collection (ANC), infected pancreatic necrosis (IPN), other local complications of pancreatitis, persistent renal failure, respiratory failure, and multiple organ failure (MOF). Outcomes assessed included 28-day and 90-day mortality, overall mortality, hospital length of stay (LOS), ICU length of stay, and use of renal replacement therapy (CRRT), mechanical ventilation, and percutaneous catheter drainage.

### Definition of oxygen partial pressure groups

Patients were classified based on their lowest recorded PaO_2_ values during the first 3 days following admission to the pancreatic ICU. If multiple arterial blood gas measurements were obtained on the same day, the lowest PaO_2_ value was selected. Patients who maintained PaO_2_ ≥ 80 mmHg on all 3 days were assigned to the liberal PaO_2_ group; all others were classified into the conservative PaO_2_ group [11].

The first ICU day was defined as the period from ICU admission until the first midnight. The second and third ICU days were defined as the subsequent 24-h intervals beginning at midnight [12].

The detection of arterial partial pressure of oxygen originates from blood gas analysis, and the Pancreatic ICU uses the GEM Premier 5000 Werfen blood gas analyzer. The manufacturer is located in the United States.

### Statistical analysis

Continuous variables are presented as mean ± standard deviation (SD) if normally distributed, or as median with interquartile range (IQR) if skewed. For missing values in the data, we first conducted a randomization test for missing data, and then excluded data sets with more than 20% missing values. Meanwhile, we used the K-nearest neighbor imputation algorithm (Knn) and multiple imputation methods to impute the data. When the baseline data of patients were unbalanced, we matched and balanced the baseline data of the two groups of patients through a propensity score matching (PSM) cohort, and calculated the absolute standardized mean difference (SMD) corresponding to each variable to check the degree of PSM and evaluate the imbalance between the two groups before and after matching. We also used inverse probability of treatment weighting (IPTW), standardized mortality ratio weighting (SMRW), pairing algorithm (PA), and overlap weighting (OW) for weighting. Finally, when the SMD value is ≤0.1, it indicates that all variables between the two groups are in a balanced state.

After balancing baseline characteristics, univariable logistic regression was performed to compare clinical outcomes—including persistent circulatory failure, multiple organ dysfunction, infected pancreatic necrosis, persistent renal failure, and persistent respiratory failure—as well as the use of mechanical ventilation and continuous renal replacement therapy (CRRT). Odds ratios (ORs) with 95% confidence intervals (CIs) were calculated to evaluate whether the incidence of organ failure was reduced. In addition, after further processing with Bonferroni correction, all *P* values are statistically significant. The proportional hazards (PH) assumption was tested and satisfied (all *P* > 0.05). Cox proportional hazards regression was used to analyze mortality outcomes, yielding hazard ratios (HRs) with 95% CIs to assess the association between higher PaO_2_ levels and mortality.

Subgroup analyses were conducted based on smoking history, age, BMI, SIRS, and APACHE II score to evaluate which groups of people benefit. Kaplan–Meier survival curves were generated to compare 28-day, 90-day, and overall mortality between groups. Sensitivity analyses were performed to test the robustness of the findings under different assumptions, including exclusion of patients with recurrent acute pancreatitis, those with chronic obstructive pulmonary disease (COPD), and inclusion only of patients admitted within 3 days of symptom onset.

All statistical analyses were conducted using R software, version 4.2.2.

## Results

### Data collection and baseline characteristics

**Fig. 1:**
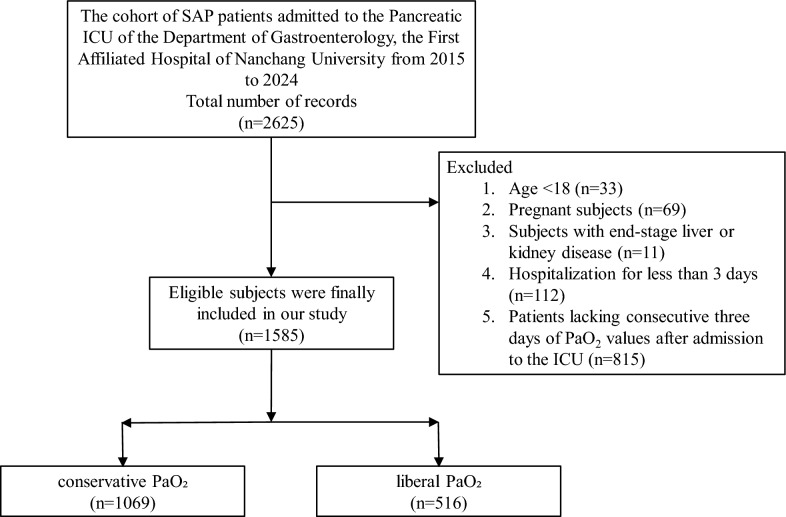
Flowchart of the screening and enrollment of eligible subjects. SAP, severe acute pancreatitis; ICU, intensive care unit

In this study, a total of 2625 SAP patients from the First Affiliated Hospital of Nanchang University were selected. After applying the exclusion criteria, 1585 patients were screened and included in the final study (Fig. 1). Meanwhile, after propensity score matching, they were divided into the conservative PaO_2_ group (1069 cases) and the liberal PaO_2_ group (516 cases). Table S1 shows a comparison of baseline data between the conservative PaO_2_ group and the liberal PaO_2_ group. In terms of age, the median age in the conservative group was 51.0 (39.0–64.0), while that in the liberal group was 46.0 (35.0–63.0). The number of females (392 vs. 181), as well as the numbers of people with a smoking history (345 vs. 177) in the conservative group, were all higher than those in the liberal group. There was no significant difference in BMI between the two groups [24.5 (22.6–27.1) vs. 24.6 (22.7–27.7)]. Except for age, TG, Ca, and pH, the other baseline data were similar. After propensity score matching and IPTW method (Table 1), the baseline data were more balanced than before matching, and the SMD value was still ≤0.1.

**Table 1:** Baseline characteristics of patients in conservative and liberal PaO_2_ groups after the IPTW matching

Characteristic	Unmatched			After IPTW matching			
	Conservative PaO_2_ (*n* = 1069)	Liberal PaO_2_ (*n* = 516)	SMD	Conservative PaO_2_ (*n* = 1069)	Liberal PaO_2_ (*n* = 516)	SMD	
*N*	1069	516		1069	516		
Sex (male), *n* (%)	677 (63.3)	335 (64.9)	0.033	683.1 (63.8)	327.7 (63.7)	0.003	<0.1
Age (years)	51.71 (16.05)	49.39 (17.82)	0.137	51.00 (16.05)	51.20 (18.05)	0.012	<0.1
BMI (kg/m^2^)	25.11 (3.82)	25.23 (4.09)	0.031	25.14 (3.87)	25.10 (4.11)	0.011	<0.1
Smoking, *n* (%)	345 (32.3)	177 (34.3)	0.043	354.3 (33.1)	172.0 (33.4)	0.007	<0.1
Drinking, *n* (%)	381 (35.6)	197 (38.2)	0.053	390.7 (36.5)	190.0 (36.9)	0.009	<0.1
Hypertension, *n* (%)	293 (27.4)	129 (25.0)	0.055	285.0 (26.6)	139.3 (27.1)	0.01	<0.1
COPD, *n* (%)	6 (0.6)	5 (1.0)	0.047	7.6 (0.7)	3.6 (0.7)	<0.001	<0.1
Diabetes, *n* (%)	161 (15.1)	94 (18.2)	0.085	171.9 (16.1)	82.9 (16.1)	0.001	<0.1
Temperature (℃)	37.26 (0.81)	37.20 (0.71)	0.084	37.23 (0.81)	37.22 (0.73)	0.014	<0.1
Pulse (bpm)	106.83 (21.58)	107.94 (22.03)	0.051	107.30 (21.76)	107.41 (22.23)	0.005	<0.1
Respirations (bpm)	26.83 (7.72)	26.28 (7.68)	0.071	26.65 (7.67)	26.60 (7.67)	0.006	<0.1
Mean arterial pressure (mmHg)	99.70 (16.19)	101.17 (16.99)	0.089	100.21 (16.21)	100.13 (16.97)	0.005	<0.1
WBC (×10^9^/L)	14.43 (6.25)	14.21 (6.28)	0.034	14.34 (6.21)	14.26 (6.29)	0.013	<0.1
HCT (%)	39.94 (9.25)	40.24 (10.07)	0.03	40.03 (9.41)	40.02 (9.79)	0.001	<0.1
ALB (g/L)	34.18 (5.94)	34.82 (6.43)	0.102	34.40 (6.14)	34.42 (6.24)	0.003	<0.1
TG (mmol/L)	6.58 (9.38)	7.73 (9.91)	0.119	7.00 (10.00)	7.00 (9.34)	0.001	<0.1
BUN (mmol/L)	8.40 (6.53)	8.05 (6.38)	0.054	8.29 (6.41)	8.30 (6.51)	0.001	<0.1
Cr (mmol/L)	123.85 (121.43)	118.75 (103.75)	0.045	121.99 (117.96)	120.94 (104.27)	0.009	<0.1
Ca (mmol/L)	1.92 (0.33)	1.96 (0.34)	0.117	1.93 (0.32)	1.93 (0.35)	0.003	<0.1
PH	7.40 (0.08)	7.38 (0.09)	0.215	7.39 (0.08)	7.39 (0.08)	0.008	<0.1
SIRS	2.32 (0.91)	2.28 (0.91)	0.042	2.31 (0.91)	2.30 (0.92)	0.011	<0.1
APACHE II	12.53 (4.94)	12.92 (5.20)	0.077	12.69 (5.06)	12.72 (5.15)	0.005	<0.1

Data are presented as mean ± standard deviation (continuous variables) or number (percentage) (categorical variables); patients in the two groups were matched using inverse probability of treatment weighting (IPTW); a standardized mean difference (SMD) < 0.1 indicates good balance between groups

*BMI* body mass index, *COPD* chronic obstructive pulmonary disease, *Bpm* beats per minute, *MAP* mean arterial pressure, *WBC* white blood cell count, *HCT* hematocrit, *ALB* albumin, *TG* triglycerides, *BUN* blood urea nitrogen, *Cr* creatinine, *Ca* calcium, *SIRS* Systemic Inflammatory Response Syndrome, *APACHEII* Acute Physiology and Chronic Health Evaluation II, *IPTW* the inverse probability of treatment weighting

### Association between oxygen partial pressure and clinical outcomes

On ICU day 1 (Table S2), the mean PaO_2_ was 119.3 ± 34.6 mmHg in the liberal group and 83.5 ± 28.5 mmHg in the conservative group. Throughout the first 3 days of ICU stay, median PaO_2_ values were significantly higher in the liberal group than in the conservative group (all *P* < 0.001).

**Fig. 2:**
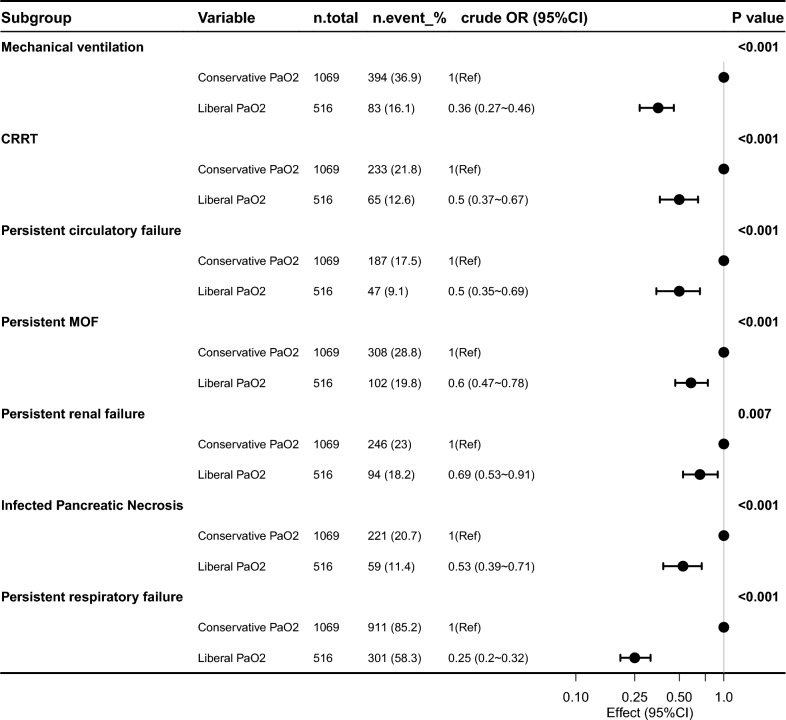
Logistic regression analysis. Association between liberal vs. conservative oxygen therapy and clinical outcomes in patients. This forest plot illustrates the results of univariable logistic regression analyses evaluating the relationship between liberal (liberal PaO_2_) and conservative (conservative PaO_2_) oxygen therapy strategies and the incidence of key clinical endpoints in patients with acute pancreatitis. Outcomes examined included mechanical ventilation, continuous renal replacement therapy (CRRT), persistent circulatory failure, persistent multiple organ failure (MOF), persistent renal failure, infected pancreatic necrosis, and persistent respiratory failure

In terms of clinical outcomes, after conducting a univariate logistic regression analysis (Fig. 2), higher PaO_2_ in the liberal group was associated with lower mortality in SAP patients. Specifically, the liberal group showed significantly lower risks of persistent circulatory failure (OR 0.50; 95% CI 0.35–0.69; *P* < 0.001), persistent renal failure (OR 0.69; 95% CI 0.53–0.91; *P* = 0.007), infected pancreatic necrosis (OR 0.53; 95% CI 0.39–0.71; *P* < 0.001), and persistent respiratory failure (OR 0.25; 95% CI 0.20–0.32; *P* < 0.001). Use of mechanical ventilation (OR 0.36; 95% CI 0.27–0.46; *P* < 0.001) and continuous renal replacement therapy (OR 0.50; 95% CI 0.37–0.67; *P* < 0.001) was also significantly reduced in the liberal group. Additional outcomes are summarized in Table 2. Compared with the conservative PaO_2_ group, the liberal PaO_2_ group had significantly shorter hospital and ICU lengths of stay (all *P* < 0.05). After inverse probability of treatment weighting (IPTW), the liberal group also exhibited significantly lower 28-day mortality (38 deaths [7.4%] vs. 130.3 deaths [12.2%]), 90-day mortality (52 deaths [10.1%] vs. 186.6 deaths [17.4%]), and overall mortality (53 deaths [10.3%] vs. 191.2 deaths [17.9%]; all *P* < 0.01), indicating better survival outcomes associated with higher PaO_2_.

**Table 2:** Comparison of clinical outcomes between the two groups after IPTW

Characteristic	Conservative PaO_2_	Liberal PaO_2_	*P* value
	1069.4	516.0	
APFC, *n* (%)	291.5 (27.3)	128.0 (24.8)	0.310
PPC, *n* (%)	9.0 (0.8)	8.0 (1.6)	0.195
WON, *n* (%)	361.5 (33.8)	145.0 (28.1)	0.025
ANC, *n* (%)	661.8 (61.9)	319.0 (61.8)	0.981
Persistent respiratory failure, *n* (%)	900.9 (84.2)	301.0 (58.3)	<0.001
Infected pancreatic necrosis, *n* (%)	211.4 (19.8)	59.0 (11.4)	<0.001
Persistent renal failure, *n* (%)	264.0 (24.7)	94.0 (18.2)	0.005
Persistent organ failure, *n* (%)	922.9 (86.3)	318.0 (61.6)	<0.001
Confirmed organ failure, *n* (%)	1037.9 (97.1)	423.0 (82.0)	<0.001
Confirmed circulatory failure, *n* (%)	194.7 (18.2)	52.0 (10.1)	<0.001
Persistent MOF, *n* (%)	315.6 (29.5)	102.0 (19.8)	<0.001
Persistent circulatory failure, *n* (%)	181.8 (17.0)	47.0 (9.1)	<0.001
Total length of hospital stay	26.21 (24.35)	22.89 (21.28)	0.007
Length of ICU stay	14.40 (15.43)	11.91 (12.88)	0.001
28-day mortality, *n* (%)	130.3 (12.2)	38.0 (7.4)	0.004
90-day mortality, *n* (%)	186.6 (17.4)	52.0 (10.1)	<0.001
Mortality, *n* (%)	191.2 (17.9)	53.0 (10.3)	<0.001
PCD, *n* (%)	245.5 (23.0)	49.0 (9.5)	<0.001
CRRT, *n* (%)	249.0 (23.3)	65.0 (12.6)	<0.001
Mechanical ventilation, *n* (%)	384.3 (35.9)	83.0 (16.1)	<0.001

*APFC* acute peripancreatic fluid collection, *PPC* pancreatic pseudocyst, *WON* walled-off necrosis, *ANC* acute necrotic collection, *MOF* multiple organ failure, *PCD* percutaneous catheter drainage, *CRRT* continuous renal replacement therapy

### Kaplan–Meier survival and cox regression analyses

**Fig. 3:**
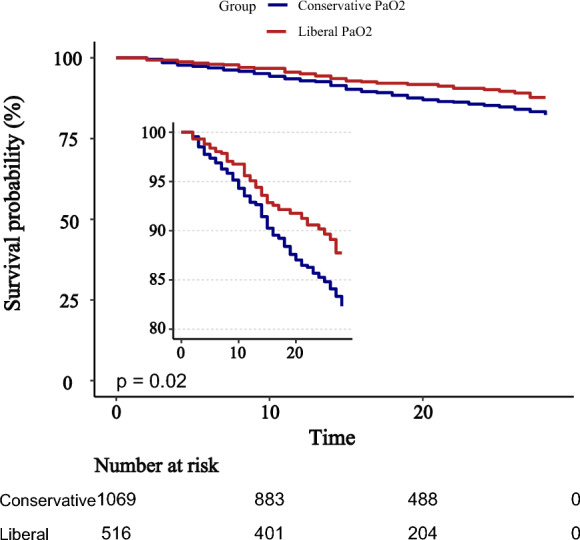
Kaplan–Meier estimates of 28-day survival in patients with severe acute pancreatitis

**Fig. 4:**
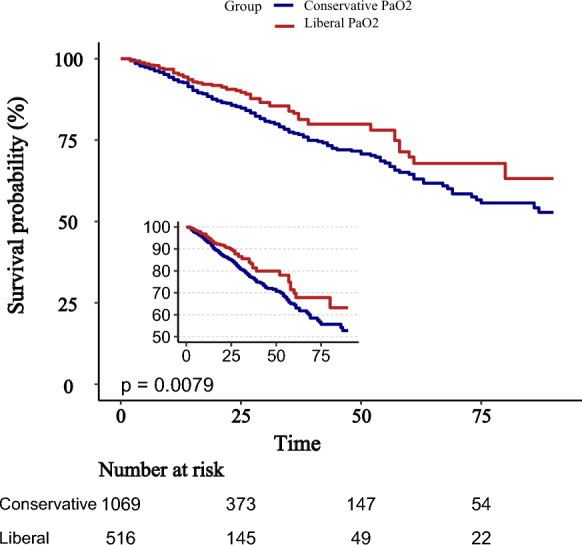
Kaplan–Meier estimates of 90-day survival in patients with severe acute pancreatitis

**Fig. 5:**
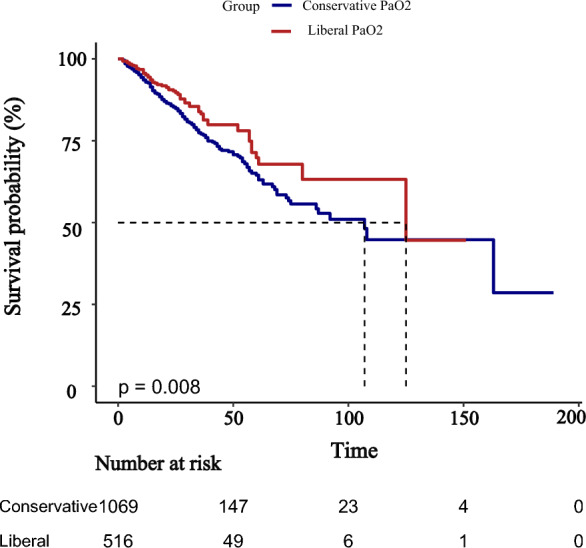
Kaplan–Meier estimates of overall survival in patients with severe acute pancreatitis

**Fig. 6:**
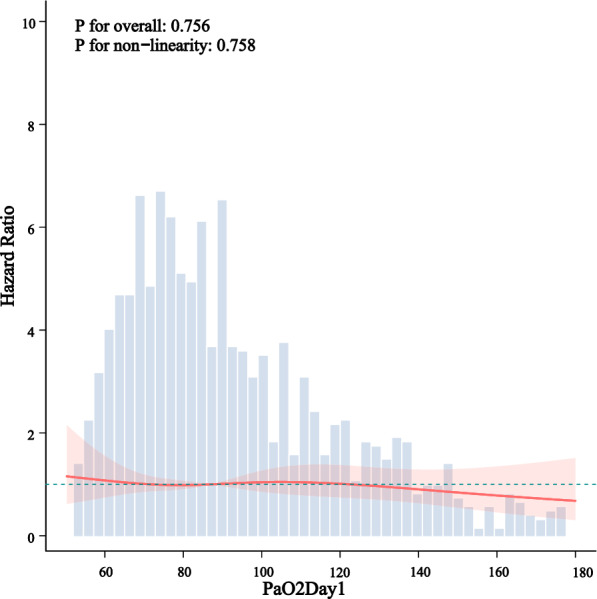
Restricted cubic spline-derived dose–response relationship between day 1 PaO_2_ levels and all-cause mortality

**Fig. 7:**
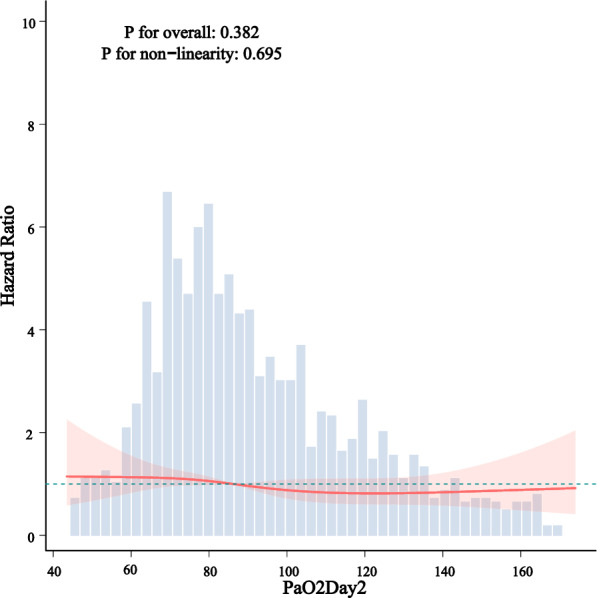
Restricted cubic spline-derived dose–response relationship between day 2 PaO_2_ levels and all-cause mortality

**Fig. 8:**
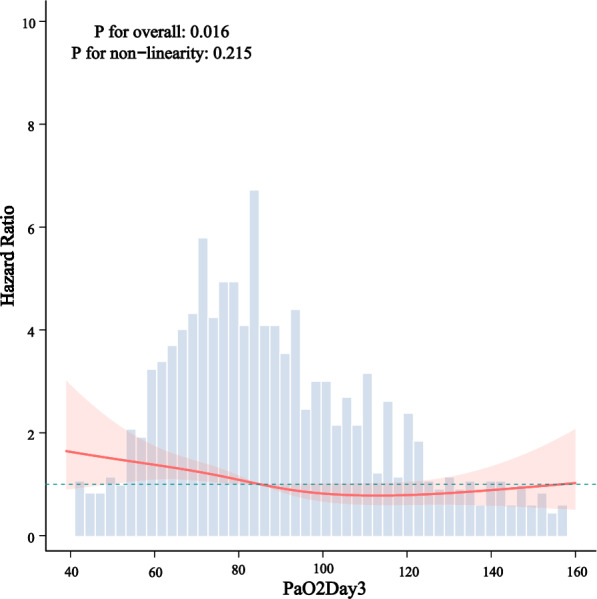
Restricted cubic spline-derived dose–response relationship between day 3 PaO_2_ levels and all-cause mortality

Kaplan–Meier survival curves demonstrated significantly higher survival probabilities in the liberal PaO_2_ group compared with the conservative group (Figs. 3, 4, 5). Cox proportional hazards regression analysis confirmed that higher PaO_2_ levels were associated with reduced mortality (Table 3), with hazard ratios of 0.67 (95% CI 0.47–0.96; *P* = 0.034) at 28 days, 0.70 (95% CI 0.52–0.94; *P* = 0.024) at 90 days, and 0.70 (95% CI 0.52–0.95; *P* = 0.025) for overall mortality, corresponding to risk reductions of 33%, 30%, and 30%, respectively. No significant association between PaO_2_ and clinical outcomes was observed during the first or second day of ICU stay (Figs. 6, 7, 8). However, by the third ICU day, higher PaO_2_ showed a significant inverse linear relationship with mortality, with the association appearing to stabilize around a PaO_2_ level of 100 mmHg.

**Table 3:** Cox analysis of death outcomes

Subgroup	n. total	n. event_%	HR (95% CI)	*P* value
28-day mortality				0.034
	1069	131 (12.3)	1 (Ref)	
	516	38 (7.4)	0.67 (0.47–0.96)	
90-day mortality				0.024
	1069	188 (17.6)	1 (Ref)	
	516	52 (10.1)	0.7 (0.52–0.94)	
Total mortality				0.025
	1069	192 (18)	1 (Ref)	
	516	53 (10.3)	0.7 (0.52–0.95)

### Subgroup and sensitivity analyses

In the subgroup analysis (Table 4), the analysis was conducted based on patients’ smoking history, age, BMI, SIRS score, and APACHE II score. There were significant differences in treatment effects among different clinically relevant subgroups in the liberal group, highlighting the key interaction between oxygen therapy effects and patient-specific pathophysiology. The results showed that patients without a smoking history (HR 0.66, 95% CI 0.45–0.95), aged < 60 years (HR 0.54, 95% CI 0.35–0.83), with BMI < 24 (HR 0.56, 95% CI 0.33–0.92), SIRS score ≥ 2 points (HR 0.69, 95% CI 0.50–0.95), and APACHE II ≥ 8 points (HR 0.65, 95% CI 0.48–0.89) were associated with a lower 28-day mortality risk.

**Table 4:** Subgroup analysis

Subgroup	Variable	n. total	28-day mortality (%)	HR (95% CI)	*P* value
*Smoking status*					
No					
	Conservative PaO_2_	724	134 (18.5)	1 (Ref)	
	Liberal PaO_2_	339	33 (9.7)	0.66 (0.45–0.95)	0.033
Yes					
	Conservative PaO_2_	345	58 (16.8)	1 (Ref)	
	Liberal PaO_2_	177	20 (11.3)	0.8 (0.48–1.31)	0.381
*Age*					
<60					
	Conservative PaO_2_	720	118 (16.4)	1 (Ref)	
	Liberal PaO_2_	371	25 (6.7)	0.54 (0.35–0.83)	0.006
≥60					
	Conservative PaO_2_	349	74 (21.2)	1 (Ref)	
	Liberal PaO_2_	145	28 (19.3)	0.85 (0.56–1.31)	0.478
*BMI*					
<24					
	Conservative PaO_2_	413	77 (18.6)	1 (Ref)	
	Liberal PaO_2_	197	17 (8.6)	0.56 (0.33–0.92)	0.029
≥24					
	Conservative PaO_2_	656	115 (17.5)	1 (Ref)	
	Liberal PaO_2_	319	36 (11.3)	0.8 (0.55–1.16)	0.255
*SIRS*					
<2					
	Conservative PaO_2_	183	24 (13.1)	1 (Ref)	
	Liberal PaO_2_	98	6 (6.1)	0.8 (0.35–1.85)	0.631
≥2					
	Conservative PaO_2_	886	168 (19)	1 (Ref)	
	Liberal PaO_2_	418	47 (11.2)	0.69 (0.5–0.95)	0.025
*APACHEII*					
<8					
	Conservative PaO_2_	158	12 (7.6)	1 (Ref)	
	Liberal PaO_2_	70	5 (7.1)	1.41 (0.53–3.7)	0.526
≥8					
	Conservative PaO_2_	911	180 (19.8)	1 (Ref)	
	Liberal PaO_2_	446	48 (10.8)	0.65 (0.48–0.89)	0.009

Sensitivity analysis (Table 5), different hypotheses were established by methods such as excluding specific populations to explore the stability of the results. It was found that the research results remained consistent and stable after establishing hypotheses by excluding patients with recurrent acute pancreatitis (HR 0.66, 95% CI 0.48–0.92, *P* = 0.019), excluding people with chronic obstructive pulmonary disease (COPD) (HR 0.64, 95% CI 0.44–0.94, *P* = 0.024), including patients admitted within 3 days of onset (HR 0.71, 95% CI 0.53–0.96, *P* = 0.031). In addition, sensitivity analysis using different weighting methods was performed on the data after propensity score matching, and after adjusting for the covariate FiO_2_ with multiple factors (Table S5). The results also showed stability.

**Table 5:** Sensitivity analysis

Subgroup	n. total	28-day mortality (%)	HR (95% CI)	*P* value
Sensitivity analysis 1				0.034
	1069	131 (12.3)	1 (Ref)	
	516	38 (7.4)	0.67 (0.47–0.96)	
Sensitivity analysis 2				0.019
	912	167 (18.3)	1 (Ref)	
	433	42 (9.7)	0.66 (0.48–0.92)	
Sensitivity analysis 3				0.024
	679	118 (17.4)	1 (Ref)	
	340	34 (10)	0.64 (0.44–0.94)	
Sensitivity analysis 4				0.031
	1063	191 (18)	1 (Ref)	
	511	53 (10.4)	0.71 (0.53–0.96)	

Sensitivity analysis 1: All patients

Sensitivity analysis 2: Excluding patients with recurrent acute pancreatitis

Sensitivity analysis 3: Excluding people with chronic obstructive pulmonary disease (COPD)

Sensitivity analysis 4: Including patients admitted to hospital within 3 days of onset

## Discussion

This retrospective cohort shows that higher PaO_2_ is associated with lower mortality in patients with SAP. Specifically, maintaining a higher PaO_2_ (≥80 mmHg) in the first 3 days after admission to the ICU is closely related to a reduced 28-day mortality rate and improved clinical outcomes. Subgroup analysis further revealed that patients aged < 60 years, with BMI < 24, SIRS score ≥ 2, APACHE II ≥ 8, and no smoking history had particularly significant survival benefits; sensitivity analysis confirmed the stability of the results. Our research findings differ from the conventional concept of conservative oxygen therapy in SAP patients and clarify the unique pathophysiological characteristics of SAP. Therefore, this study indicates that higher PaO_2_ is associated with a lower incidence of organ failure (especially AHRF) and lower mortality.

The optimal oxygen targets in critically ill patients remain controversial, with considerable variation across studies. For instance, a post-hoc analysis of the ALBIOS trial, which defined hyperoxia as PaO_2_ > 100 mmHg and normoxia as PaO_2_ ≤ 100 mmHg, found no significant difference in 28-day mortality or long-term survival among patients with sepsis [13]. In contrast, another study reported significantly lower 28-day mortality and 90-day mortality among patients with hyperoxia (PaO_2_ > 80 mmHg) during the first 3 days of ICU stay [11]. Although hypoxemia has traditionally been defined as PaO_2_ ≤ 60 mmHg, and lung-protective ventilation strategies often target PaO_2_ between 60 and 80 mmHg [14]. Although this target appears reasonable for patients with preserved oxygen delivery and utilization in tissues [3]. However, recent studies suggest that higher PaO_2_ may improve survival in conditions, such as sepsis, traumatic brain injury, and acute respiratory distress syndrome (ARDS), with ideal oxygenation targets varying by disease [11, 15–17]. This non-random therapeutic effect that occurs due to differences in treatment subjects is called treatment heterogeneity, the need to tailor oxygen therapy based on patient-specific and disease-specific characteristics [18, 19]. Based on the above theory, our research results are also consistent.

The survival benefit observed with higher PaO_2_ in SAP may be attributed to mitigation of early inflammatory cascades, microcirculatory dysfunction, and mitochondrial impairment—hallmarks of SAP pathophysiology [20]. The initial pancreatic injury triggers the release of proteases (e.g., trypsin and elastase) and lipases (e.g., phospholipase A_2_), which damage pulmonary endothelium, increase alveolar–capillary permeability, and promote intrapulmonary shunting, ultimately impairing oxygen diffusion [21–24]. SIRS often presents early in SAP, disrupts cellular oxygen utilization, and contributes to AHRF. Animal studies have implicated calcium overload and oxidative stress in amplifying inflammation [25]. Hypoxia stimulates reactive oxygen species (ROS) production and calcium release, activating inflammatory pathways (e.g., AP-1, STAT3, and MAPK) and increasing cytokines, such as IL-6, TNF-α, and IL-1 [26–28], thereby exacerbating airway inflammation and lung ischemia–reperfusion injury. Concurrently, mitochondrial apoptosis pathways are activated through cytochrome c release and opening of the mitochondrial permeability transition pore (MPTP), leading to loss of membrane potential, uncoupled respiration, and impaired ATP synthesis [29–32]. Further pancreatic necrosis releases damage-associated molecular patterns (DAMPs), which activate immune cells and amplify the inflammatory cascade [33, 34]. Necrotic acinar cells also promote procoagulant activity, contributing to microthrombosis and microcirculatory failure, characterized by reduced functional capillary density, impaired tissue perfusion, and diminished oxygen delivery [35, 36].

Consistent with these mechanisms, our subgroup analyses showed that patients with heightened systemic inflammation (SIRS ≥ 2, APACHE II ≥ 8) benefited most from higher PaO_2_. Improved oxygenation may help overcome diffusion barriers, restore mitochondrial function, and mitigate pancreatic necrosis and microvascular dysfunction—key factors in preventing AHRF [37]. The protective association persisted across multiple sensitivity analyses, supporting the robustness of our findings. Moreover, the most pronounced benefit was observed on ICU day 3 at a PaO_2_ level around 100 mmHg, suggesting a critical time window for oxygen therapy. We hypothesize that the initial 2 days represent a phase of escalating inflammation, where compensatory mechanisms remain active, whereas day 3 marks the peak of systemic inflammation and oxidative stress, during which aggressive oxygenation was most strongly associated with reduced organ failure and mortality. Whether maintaining high PaO_2_ beyond this hyperinflammatory phase remains beneficial warrants further investigation.

## Limitations

Several limitations should be acknowledged. First, despite statistical adjustments and sensitivity analyses, due to the observational nature of the study and the lack of relevant data, such as invasive/non-invasive mechanical ventilation status, duration of ventilation, positive end-expiratory pressure (PEEP), there may be residual confounding factors. Future prospective studies are needed for verification. Second, this was a single-center study, and differences in ICU practices may limit the generalizability of the findings. Third, although traditional subgroup analyses were conducted, more advanced methods such as machine learning may better account for effect heterogeneity and individual treatment responses. Fourth, this study only obtained data from the first 3 days of patients’ ICU admission and did not control for subsequent interventions and outcomes, which limits the conclusions regarding long-term management. Fifth, this study only verified whether higher arterial partial pressure of oxygen is associated with a lower mortality rate in patients with severe acute pancreatitis and failed to elaborate on the mechanism underlying the higher PaO_2_ in the liberal oxygen therapy group. Finally, observational studies may introduce time-dependent confounding factors, and it cannot be ruled out that unrecognized deterioration of circulatory or respiratory function during this period led to a decrease in PaO_2_, thereby resulting in patients being assigned to the conservative oxygen therapy group. Although we adjusted for baseline vital signs, these adjustments may not fully reflect the dynamic physiological deterioration that occurred before grouping. This requires more in-depth research to confirm.

## Conclusion

This study suggests that maintaining a higher PaO_2_ (≥80 mmHg) during the first 3 days of ICU admission was associated with lower mortality in patients with severe acute pancreatitis.

## Supplementary Information

Additional file 1.

Additional file 2.

Additional file 3.

Additional file 4.

Additional file 5.

## Data Availability

Not applicable.

## Abbreviations

AP: Acute pancreatitis
SAP: Severe acute pancreatitis
AHRF: Acute hypoxemic respiratory failure
ARDS: Acute Respiratory Distress Syndrome
COPD: Chronic obstructive pulmonary disease
ICU: Intensive care unit
SIRS: Systemic Inflammatory Response Syndrome
MAP: Mean arterial pressure
APFC: Acute peripancreatic fluid collection
WON: Walled-off necrosis
ANC: Acute necrotic collection
IPN: Infected pancreatic necrosis;
HCT: Hematocrit
ALB: Plasma albumin
APACHE II: Acute Physiology and Chronic Health Evaluation II
WBC: White blood cell count
ALB: Albumin
TG: Triglycerides
Cr: Creatinine
BUN: Blood urea nitrogen
Ca: Calcium
Lac: Lactate
BMI: Body mass index
MOF: Multiple organ failure
LOS: Hospital length of stay
IPTW: Including inverse probability of treatment weighting
SMRW: Standardized mortality ratio weighting
PA: Pairwise algorithm
OW: Overlap weighting
