# Supplementary material for: The association of arterial partial oxygen pressure with mortality in patients with severe acute pancreatitis: a retrospective cohort study
Source: Intensive Care Med Exp. 2025 Dec 18;13:131. doi: 10.1186/s40635-025-00843-8 (PMC12711611; doi:10.1186/s40635-025-00843-8)
Supplement: Supplementary file 1 — Additional file 1. [file 40635_2025_843_MOESM1_ESM.docx]

**Table S1.** Baseline characteristics of the conservative PaO_2_ and the liberal PaO_2_ groups.

| **Variable** | **Total**  **(n=1585)** | **Conservative PaO_2_**  **(n=1069)** | **Liberal PaO_2_**  **(n=516)** | ***P* Value** |
| --- | --- | --- | --- | --- |
| Age (years) | 50.0 (37.0-64.0) | 51.0 (39.0-64.0) | 46.0 (35.0-63.0) | 0.002 |
| Female, n (%) | 573 (36.2) | 392 (36.7) | 181 (35.1) | 0.536 |
| BMI, (kg/m²) | 24.5 (22.6-27.3) | 24.5 (22.6-27.1) | 24.6 (22.7-27.7) | 0.262 |
| Smoking, n (%) |  |  |  | 0.421 |
| No | 1063 (67.1) | 724 (67.7) | 339 (65.7) |  |
| Yes | 522 (32.9) | 345 (32.3) | 177 (34.3) |  |
| Drinking, n (%) |  |  |  | 0.325 |
| No | 1007 (63.5) | 688 (64.4) | 319 (61.8) |  |
| Yes | 578 (36.5) | 381 (35.6) | 197 (38.2) |  |
| Hypertension, n (%) |  |  |  | 0.309 |
| No | 1163 (73.4) | 776 (72.6) | 387 (75) |  |
| Yes | 422 (26.6) | 293 (27.4) | 129 (25) |  |
| COPD, n (%) |  |  |  | 0.351 |
| No | 1574 (99.3) | 1063 (99.4) | 511 (99) |  |
| Yes | 11 (0.7) | 6 (0.6) | 5 (1) |  |
| History of diabetes, n (%) |  |  |  | 0.109 |
| No | 1330 (83.9) | 908 (84.9) | 422 (81.8) |  |
| Yes | 255 (16.1) | 161 (15.1) | 94 (18.2) |  |
| Temperature, °C | 37.1 (36.7-37.8) | 37.1 (36.6-37.8) | 37.0 (36.7-37.6) | 0.337 |
| Pulse, (bpm) | 107.0 (90.0-122.0) | 106.0 (90.0-122.0) | 108.0 (92.0-122.2) | 0.470 |
| Mean arterial pressure, (mmHg) | 100.1 (16.5) | 99.7 (16.2) | 101.1 (17.0) | 0.108 |
| Respirations, (bpm) | 25.0 (20.0-31.0) | 26.0 (20.0-31.0) | 25.0 (20.0-31.0) | 0.223 |
| WBC (×10^9^/L) | 13.2 (10.0-18.0) | 13.4 (10.0-18.0) | 13.0 (9.6-17.7) | 0.277 |
| HCT (%) | 40.8 (34.1-47.0) | 40.5 (34.1-46.4) | 41.2 (34.0-47.8) | 0.373 |
| ALB (g/L) | 34.0 (30.1-38.6) | 33.8 (30.1-38.2) | 34.7 (30.3-39.3) | 0.093 |
| TG (mmol/L) | 2.4 (1.2-9.0) | 2.3 (1.2-8.1) | 3.1 (1.3-10.9) | 0.016 |
| BUN (mmol/L) | 6.5 (4.3-10.0) | 6.5 (4.4-10.0) | 6.5 (3.9-9.8) | 0.195 |
| Cr (mmol/L) | 79.2 (58.5-133.4) | 78.3 (58.0-131.9) | 79.9 (59.0-137.0) | 0.699 |
| Ca (mmol/L) | 2.0 (1.7-2.1) | 2.0 (1.7-2.1) | 2.0 (1.8-2.2) | 0.022 |
| PH | 7.4 (7.3-7.4) | 7.4 (7.4-7.4) | 7.4 (7.3-7.4) | <0.001 |
| SIRS | 2.0 (2.0-3.0) | 2.0 (2.0-3.0) | 2.0 (2.0-3.0) | 0.512 |
| APACHE II | 12.0 (9.0-16.0) | 12.0 (9.0-16.0) | 12.0 (9.0-16.0) | 0.164 |

Abbreviations as in Table 1.

**Table S2.** Distribution of patients according to PaO_2_ values for the first three days in the ICU.

| **Variables** | **Total**  **(n = 1585)** | **Conservative PaO_2_**  **(n = 1069)** | **Liberal PaO_2_**  **(n = 516)** | ***P* value** |
| --- | --- | --- | --- | --- |
| Day1 PaO_2_, (Mean ± SD) | 95.2 ± 34.9 | 83.5 ± 28.5 | 119.3 ± 34.6 | < 0.001 |
| Day2 PaO_2_, (Mean ± SD) | 93.9 ± 33.2 | 81.5 ± 26.3 | 119.6 ± 31.2 | < 0.001 |
| Day3 PaO_2_, (Mean ± SD) | 90.3 ± 31.2 | 79.5 ± 27.0 | 112.7 ± 27.2 | < 0.001 |

ICU, Intensive Care Unit.

**Table S3.** Hazard Ratios (HR) and 95%CI for the Association between Liberal PaO_2_ and Conservative PaO_2_under Different Adjustment Methods.

| **Models** | **Variable** | **HR (95% CI)** | ***P* value** |
| --- | --- | --- | --- |
| Unmatched, crude | Liberal PaO_2_ | 0.66 (0.49 - 0.9) | 0.009 |
| Multivariable - adjusted | Liberal PaO_2_ | 0.65 (0.48 - 0.88) | 0.006 |
| Propensity Score - adjusted | Liberal PaO_2_ | 0.68 (0.5 - 0.92) | 0.013 |
| Propensity Score - Matched | Liberal PaO_2_ | 0.68 (0.49 - 0.96) | 0.029 |
| Weighted, IPTW | Liberal PaO_2_ | 0.7 (0.52 - 0.95) | 0.025 |
| Weighted, SMRW | Liberal PaO_2_ | 0.66 (0.49 - 0.9) | 0.009 |
| Weighted, PA | Liberal PaO_2_ | 0.67 (0.48 - 0.94) | 0.011 |
| Weighted, Ow | Liberal PaO_2_ | 0.68 (0.45 - 1.03) | 0.014 |

This table shows hazard ratios (HRs) and 95% confidence intervals (CIs) across different models. Unmatched crude model is unadjusted; multivariable - adjusted includes covariates. Propensity score - adjusted/matched use propensity scores, while weighted models (IPTW, SMRW, PA, Ow) apply propensity - based weights. HRs reflect relative risk over time; 95% CIs indicate true HR range. P < 0.05 is statistically significant.

**Table S4.** Comparison of baseline characteristics between two groups after propensity score matching with supplementary FiO_2_.

|  | After IPTW Matching | | | |
| --- | --- | --- | --- | --- |
| Characteristic | Conservative PaO_2_ (n = 1069) | Liberal PaO_2_ (n = 516) | SMD |  |
| n | 1171.6 | 450.1 |  |  |
| Sex (Male), n (%) | 774.0 (66.1) | 293.4 (65.2) | 0.019 | <0.1 |
| Age (years) | 50.51 (16.28) | 50.26 (17.47) | 0.014 | <0.1 |
| BMI, (kg/m²) | 25.09 (4.05) | 25.22 (4.16) | 0.033 | <0.1 |
| Smoking, n (%) | 385.7 (32.9) | 149.0 (33.1) | 0.004 | <0.1 |
| Drinking, n (%) | 446.3 (38.1) | 163.7 (36.4) | 0.036 | <0.1 |
| Hypertension, n (%) | 322.2 (27.5) | 126.1 (28.0) | 0.011 | <0.1 |
| COPD, n (%) | 6.3 ( 0.5) | 3.0 ( 0.7) | 0.016 | <0.1 |
| Diabetes, n (%) | 201.8 (17.2) | 78.4 (17.4) | 0.005 | <0.1 |
| Temperature, ℃ | 37.22 (0.81) | 37.23 (0.72) | 0.008 | <0.1 |
| Pulse, (bpm) | 107.09 (21.64) | 108.06 (21.64) | 0.045 | <0.1 |
| Respirations, (bpm) | 26.14 (7.63) | 26.58 (7.56) | 0.059 | <0.1 |
| MAP, (mmHg) | 99.88 (16.59) | 100.22 (16.51) | 0.021 | <0.1 |
| WBC (×10^9^/L) | 14.17 (6.06) | 14.21 (6.23) | 0.006 | <0.1 |
| HCT (%) | 39.92 (10.02) | 40.58 (9.98) | 0.066 | <0.1 |
| ALB (g/L) | 34.59 (6.11) | 34.79 (6.81) | 0.031 | <0.1 |
| TG (mmol/L) | 7.29 (10.86) | 7.62 (10.26) | 0.032 | <0.1 |
| BUN (mmol/L) | 9.01 (8.27) | 8.71 (6.64) | 0.041 | <0.1 |
| Cr (mmol/L) | 135.92 (156.32) | 133.93 (119.94) | 0.014 | <0.1 |
| Ca (mmol/L) | 1.94 (0.34) | 1.93 (0.37) | 0.033 | <0.1 |
| PH | 7.39 (0.09) | 7.38 (0.08) | 0.049 | <0.1 |
| SIRS | 2.30 (0.93) | 2.32 (0.92) | 0.026 | <0.1 |
| APACHE II | 12.66 (5.09) | 12.76 (5.21) | 0.019 | <0.1 |
| Fi0_2_ (%) | 31.57 (16.98) | 31.62 (9.49) | 0.004 | <0.1 |

Abbreviations as in Table 1.

**Table S5.** Adjusted model after adding FiO_2_ as a covariate in the logistic multifactorial analysis.

| **Variable** | **Crude.OR**  **(95% CI)** | ***P* value** | **Adjusted OR (95% CI)** | ***P* value** |
| --- | --- | --- | --- | --- |
| Conservative PaO_2_ | 1(Ref) |  | 1(Ref) |  |
| Liberal PaO_2_ | 0.52 (0.38~0.72) | <0.001 | 0.51 (0.37~0.7) | <0.001 |
