# Supplementary figures and images for: The association of arterial partial oxygen pressure with mortality in patients with severe acute pancreatitis: a retrospective cohort study
Source: Intensive Care Med Exp. 2025 Dec 18;13:131. doi: 10.1186/s40635-025-00843-8 (PMC12711611; doi:10.1186/s40635-025-00843-8)

PS distribution by treatment

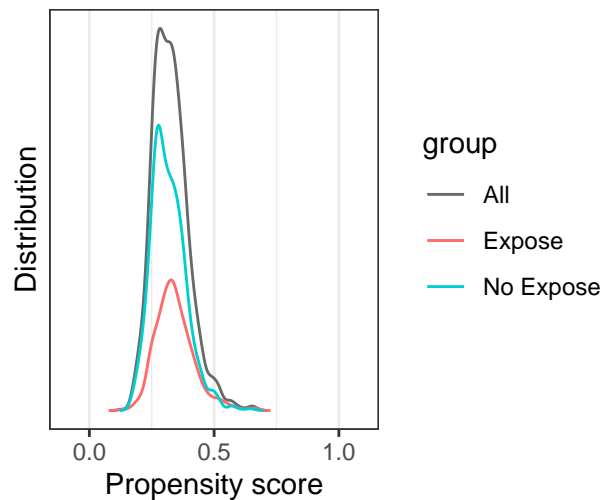

Matched Data

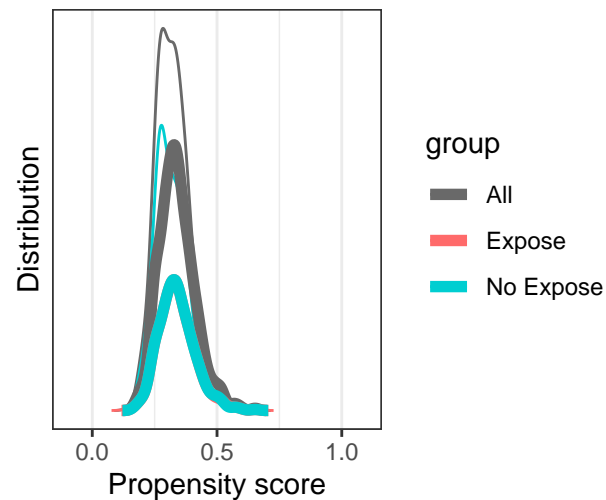

IPTW

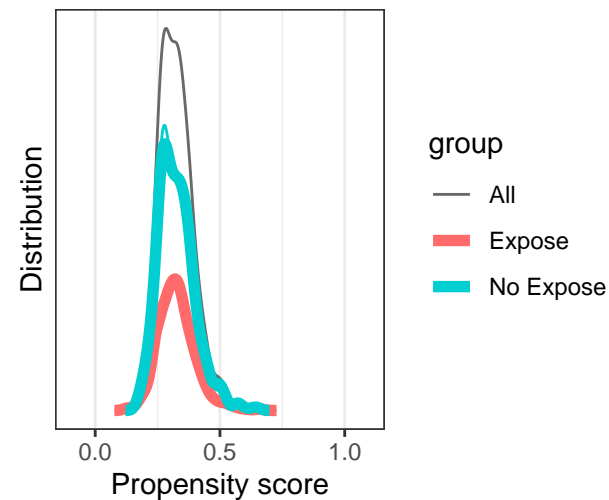

SMRW

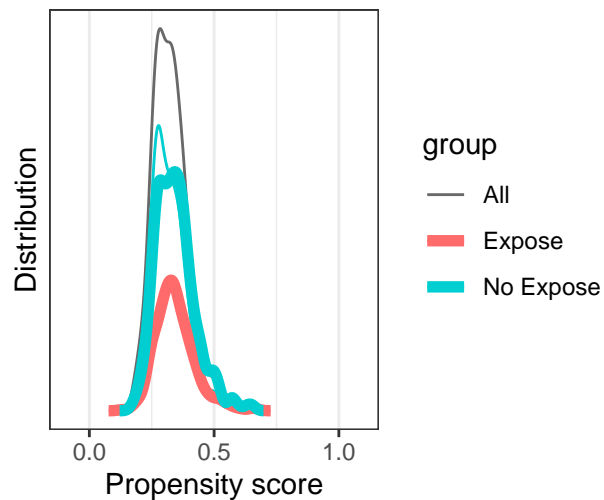

PA

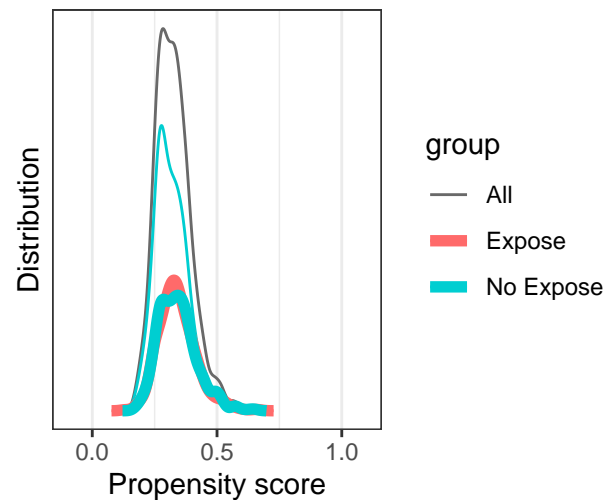

OW

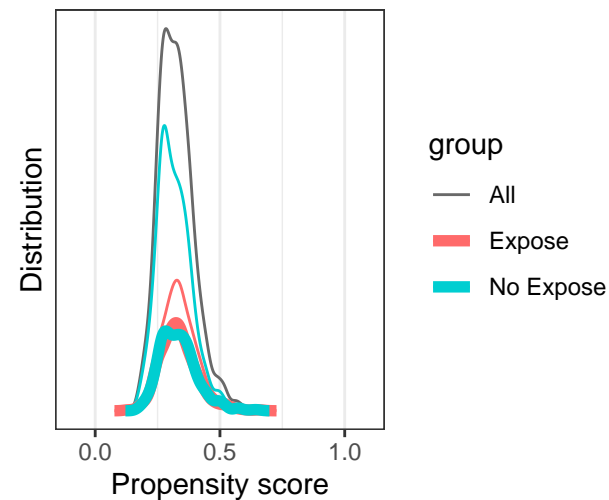

Supplement: Supplementary file 3 — Additional file 3. [file 40635_2025_843_MOESM3_ESM.pdf]

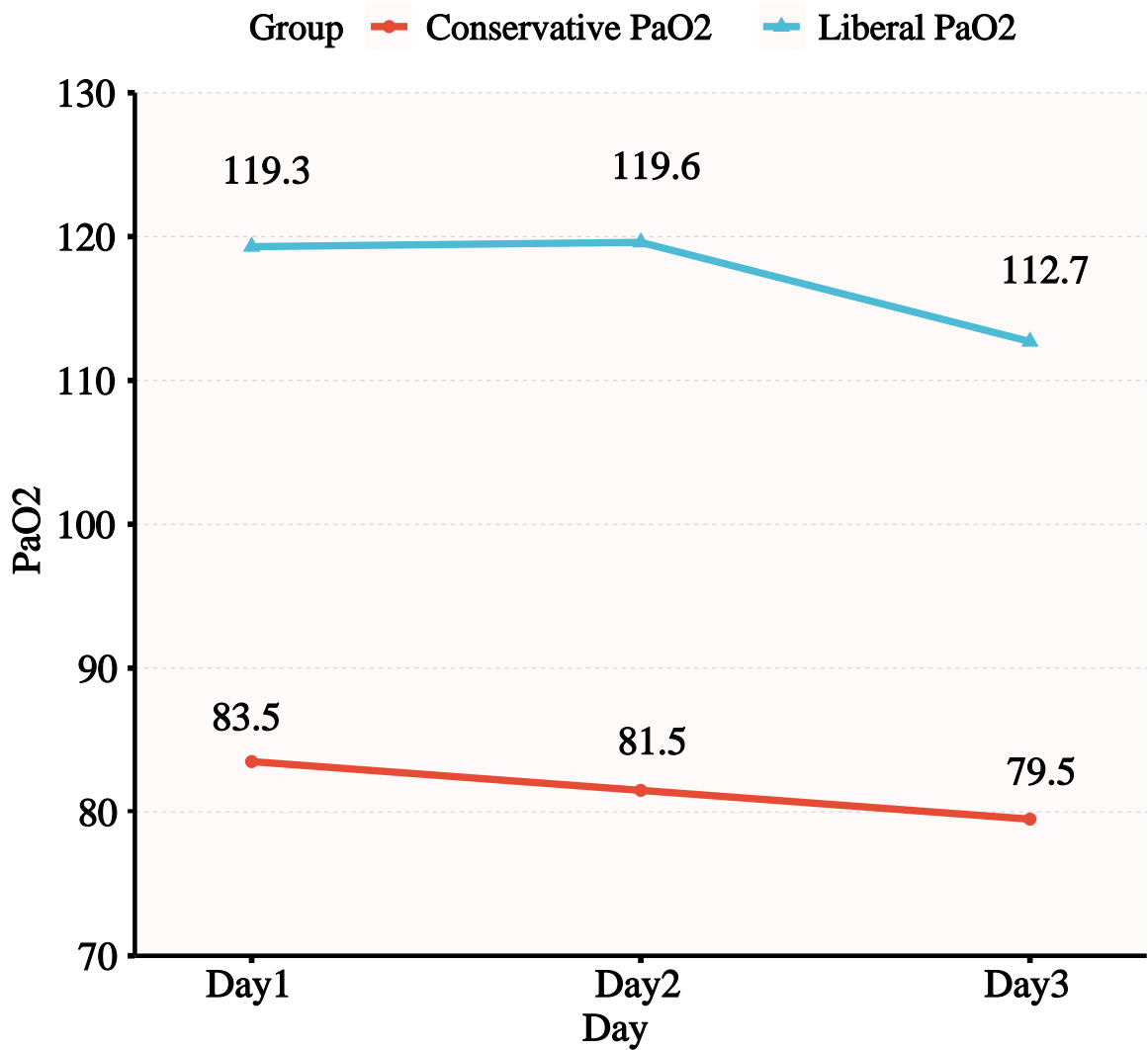

Supplement: Supplementary file 4 — Additional file 4. [file 40635_2025_843_MOESM4_ESM.pdf]

Beta(t) for Conservative PaO2

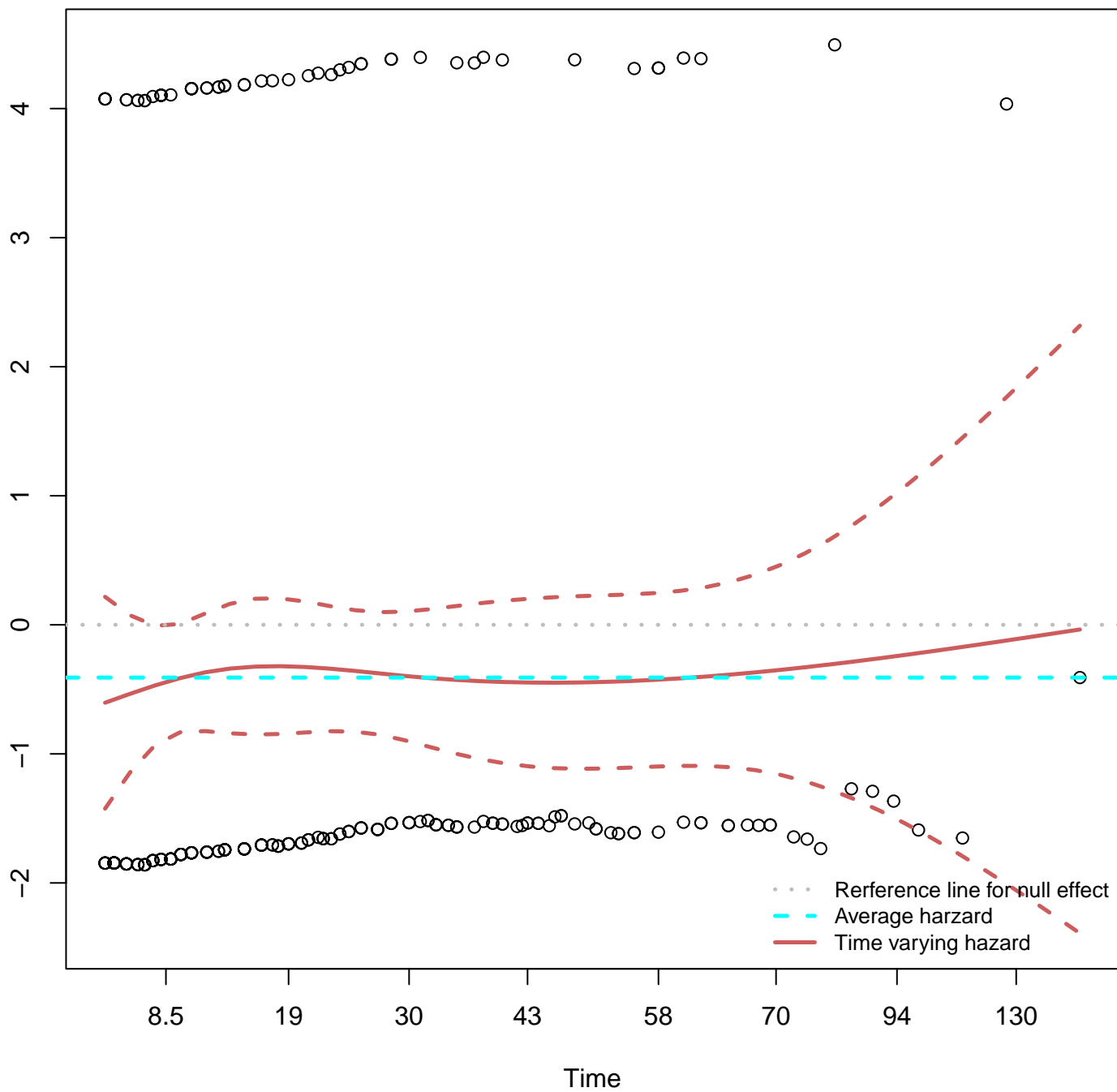

Supplement: Supplementary file 5 — Additional file 5. [file 40635_2025_843_MOESM5_ESM.pdf]
