# Supplementary material for: The association of arterial partial oxygen pressure with mortality in patients with severe acute pancreatitis: a retrospective cohort study
Source: Intensive Care Med Exp. 2025 Dec 18;13:131. doi: 10.1186/s40635-025-00843-8 (PMC12711611; doi:10.1186/s40635-025-00843-8)
Supplement: Supplementary file 2 — Additional file 2. [file 40635_2025_843_MOESM2_ESM.pdf]

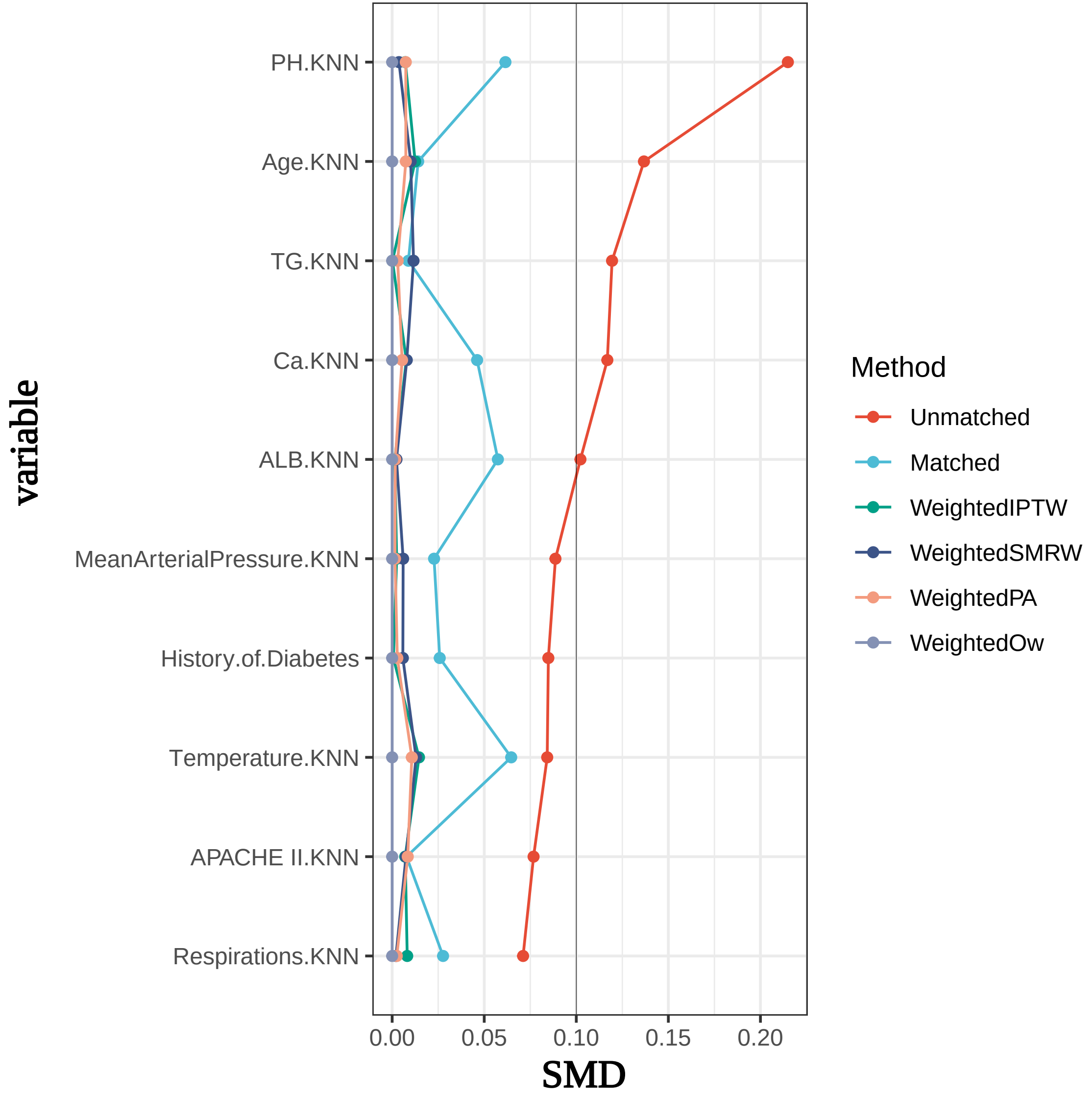
variable
PH.KNN
Age.KNN
TG.KNN
Ca.KNN
ALB.KNN
MeanArterialPressure.KNN
History.of.Diabetes
Temperature.KNN
APACHE II.KNN
Respirations.KNN
0.00
0.05
0.10
0.15
0.20
SMD
Method
Unmatched
Matched
WeightedIPTW
WeightedSMRW
WeightedPA
WeightedOw
